# Socioeconomic Status Association With Dependency From Objective and Subjective Assessments: A Cross-Sectional Study

**DOI:** 10.3389/fpsyt.2022.898686

**Published:** 2022-06-29

**Authors:** YiYang Pan, Ayizuhere Aierken, XiWen Ding, Yuan Chen, Ying Li

**Affiliations:** Department of Social Medicine, School of Public Health, Zhejiang University, Hangzhou, China

**Keywords:** dependency, socioeconomic status, social resources, objective and subjective assessments, elderly people

## Abstract

**Background:**

The effect of socioeconomic status (SES) on dependency is still complex and not fully clear. The purposes of this study are to assess the association between SES and dependency personality disorder (DPD) using both objective and subjective assessments.

**Methods:**

A cross-sectional study was conducted in 27 locations in China among 1,276 general residents aged 60 years and above through a complex multistage sampling design. Data were collected using a questionnaire by well-trained investigators through face-to-face interviews. The DPD was assessed using a standardized Chinese version of the Minnesota Multiphasic Personality Inventory-II scale. Objective SES was assessed by the combination of education levels, individual income, preretirement occupation, and medical insurance. Subjective SES was measured using the MacArthur Scale. The logistic regression analysis was used to evaluate the association between objective SES and DPD. Analysis of covariance was conducted to compare the mean of DPD scores in different levels of SES.

**Results:**

The results of the chi-squared test showed that the levels of objective SES were associated with DPD, depression, social resources, and region. The logistic regression analysis showed a significant negative association between the levels of objective SES and DPD. The odds ratio was 1.84 (95% confidence interval, 1.07–3.18) after adjusting for important confounding factors. The analysis of covariance showed differences in the mean of DPD scores among different groups defined by different levels of SES.

**Conclusion:**

The levels of SES were negatively associated with DPD, and subjective SES had a stronger association with DPD than objective SES. The effect of subjective SES on DPD is possibly associated with the perception of position in the social hierarchy.

## Introduction

With economic and social development, the increasing number of elderly people worldwide results in the increasing challenge of health and social care demand in the next few decades (1, 2). Owing to the general reductions in social and economic resources and physical function decline, dependency is generally regarded as the inevitable result of aging and has become an important public health problem (3).

Dependency is a personality disorder, and its primary feature was identified as “a pervasive and excessive need to be taken care of, or meet their emotional and physical needs which lead to the gradual loss of autonomy and clinging behavior.” Dependency has historical roots far preceding the seminal volume of the *Diagnostic and Statistical Manual of Mental Disorders* (DSM) (4, 5).

The different dependency objects, such as substances, behaviors, and people, can lead to alcohol dependency, sleep dependency, and nursing dependency. Dependency personality disorder (DPD) manifests differently at various points in a human life span; in children, it is characterized by helplessness, indecisiveness, and a tendency to cling to a supportive parent. In adolescents, it may manifest as close relationships with valued peers rather than with their parents. As the individual changes from adolescence to adulthood, the primary object of dependency may change again from peers to a mentor or figure of authority. The elderly with DPD often show psychopathological symptoms, such as loss of motivation, feelings of loneliness, and a sense of helplessness, which can bring about severe depressive symptoms and other health-related problems (6). In old age, with the impairment of physical function, cognitive decline, and the lack of social and environmental resources, the objects that cause dependency among elderly people become increasingly complex. Those elderly people with disability and cognitive impairment are associated with increased length of hospital stay and dependency for caregivers. Other older people with low income are financially dependent on their adult children. The appearance of dependent behaviors seems to be an adaptive response to debilitating socioeconomic circumstances. Epidemiological studies have confirmed that a high level of dependency is related to the risk of nutritional deficiency, depression, suicide, and increased all-cause mortality (7–9). In addition, high levels of dependency are associated with excessive use of healthcare services and increased healthcare expenditures, thus giving rise to medical burdens. DPD may have a negative impact on life satisfaction, as societal costs of increasing dependency increase over time (10).

A previous study has shown that socioeconomic factors, such as gender inequality, residence, age trends, and occupations, have a significant predictive power for an impending onset of dependency (11). A longitudinal study conducted on elderly people in Taiwan that has shown subjective socioeconomic status (SES) assessments seems to be more favorable than the objective SES assessment as a predictor of health outcomes (12). Subjective social status (SSS) can capture more comprehensive and dynamic attributes of SES than objective SES (13). Subjective SES reflects the relative rather than the absolute status in the social hierarchy. In addition, the perception of subordinate status in the social hierarchy is believed to have a destructive effect on health outcomes (14).

To the best of our knowledge, the impact of SES on dependency is understudied, particularly for subjective SES. The main objectives of this study include assessing the association between DPD and SES, using both objective and subjective measurements, and further determining the modifiable risk factors for future intervention studies.

## Materials and Methods

### Study Design

The data for this study were drawn from the project titled, “Accessibility Evaluation of Health-related Resources for the Elderly” using a cross-sectional design. Informed consent was obtained from each participant before participation. This study was approved by the institutional review board at the School of Medicine, Zhejiang University.

### Participants

**Figure 1:**
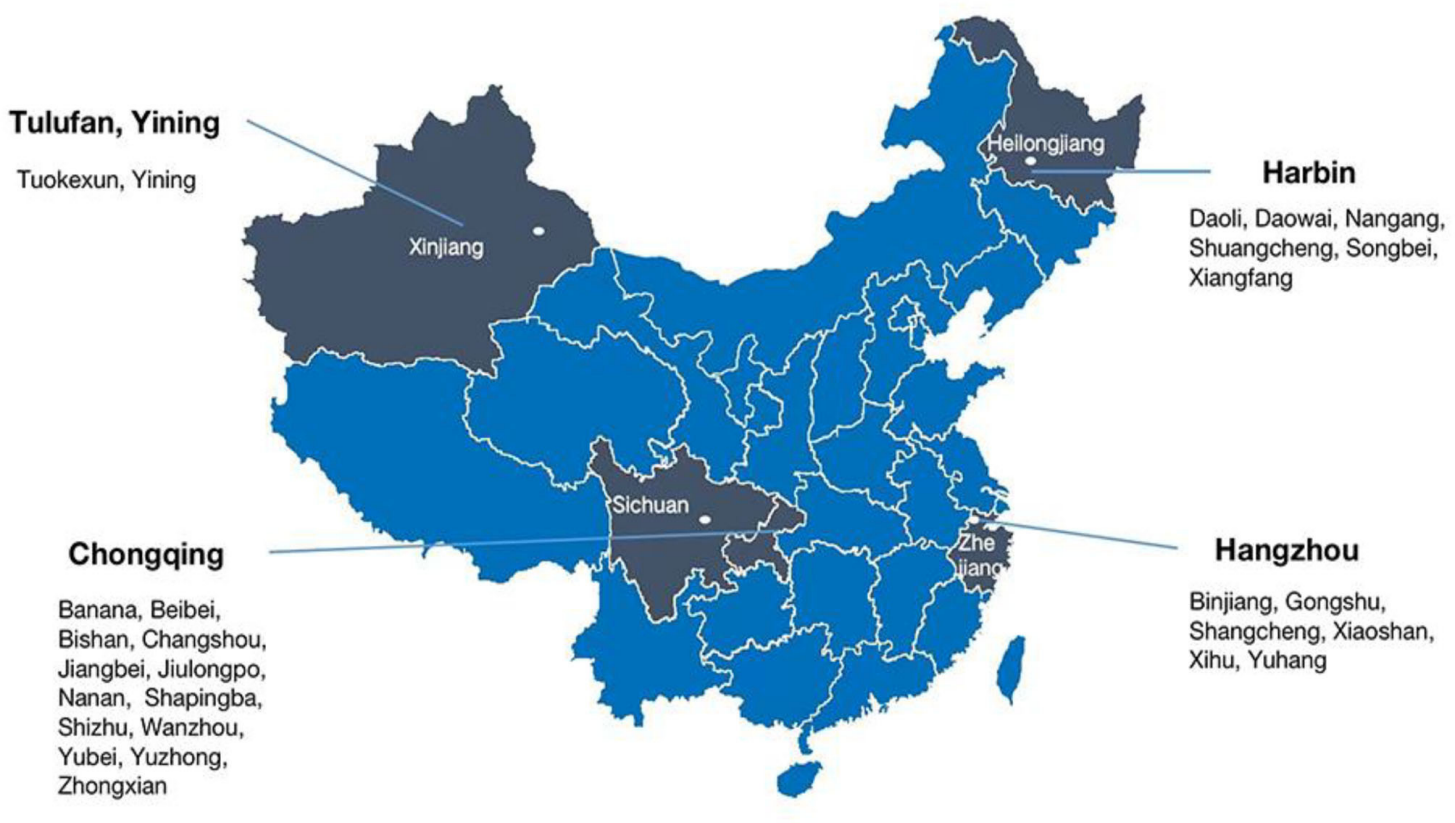
Sampling designs in the study.

A total of 1,276 general residents aged 60 years and above were selected using a complex multistage sampling design. According to the geographical distribution of China, sampling was conducted in four provinces (Zhejiang, Heilongjiang, Xinjiang, and Sichuan) from July 2019 to September 2021. In total, 27 locations (urban/rural) with good managerial and organizational capabilities were selected from five cities (Hangzhou, Harbin, Tulufan, Yining, and Chongqing), and Figure 1 shows the third sampling units. We computed the total sample size required based on the events per variable method. Potentially eligible participants were recruited through mobile phones by local staff members, followed by extensive publicity campaigns.

Among them, 271 participants were interviewed about the subjective SES using the SSS Scale. Participants who failed to complete the questionnaire were excluded.

### Data Collection

Data were collected on-site by well-trained investigators through face-to-face interviews to ensure true answers. The duration of each interview lasted 45–60 min or longer. The questionnaire consisted of fifteen parts comprising 520 items. The main content comprised demographic characteristics, psychological status, cognitive status, resource utilization, and personality.

### Measurements

#### Objective Socioeconomic Status

We combined the following measurements as a comprehensive measurement of objective SES: education level, individual income, pre-retirement occupation, and medical insurance. Education level was measured as completed years of schooling (0, ≤ 6, 7–9, 10–12, 13–17, and ≥18 years) with a value of 0–5, respectively. Individual income was measured by self-reported monthly income, which was divided into < ¥2,000, 2,000–3,999, 4,000–5,999, 6,000–7,999, and more than ¥8,000, ranging from 0 to 4, respectively. The preretirement occupation was classified with reference to the empirical study (15), which were divided into two categories with values of 0 and 1 according to manual (unemployed workers, temporary workers, factory workers, transportation personnel, housework, and labor in agriculture, fishery, and animal husbandry) and non-manual (staff of state agencies and institutions, service and sales workers, medical and health personnel, educators, and self-employed). Medical insurance was categorized into rural cooperative medical care, social basic medical insurance, and free medical care, each assigned a value of 1–3 points. If participants had commercial health insurance, an additional point would be added. 0 represents those with no health insurance. The objective SES scores ranged from 0 to 13 points; the higher the score, the higher the objective SES. In addition, it was divided into two categories with a cutoff point value of 3 based on quartiles (50th percentile), such as low objective SES and high objective SES (16).

#### Subjective Socioeconomic Status

**Figure 2:**
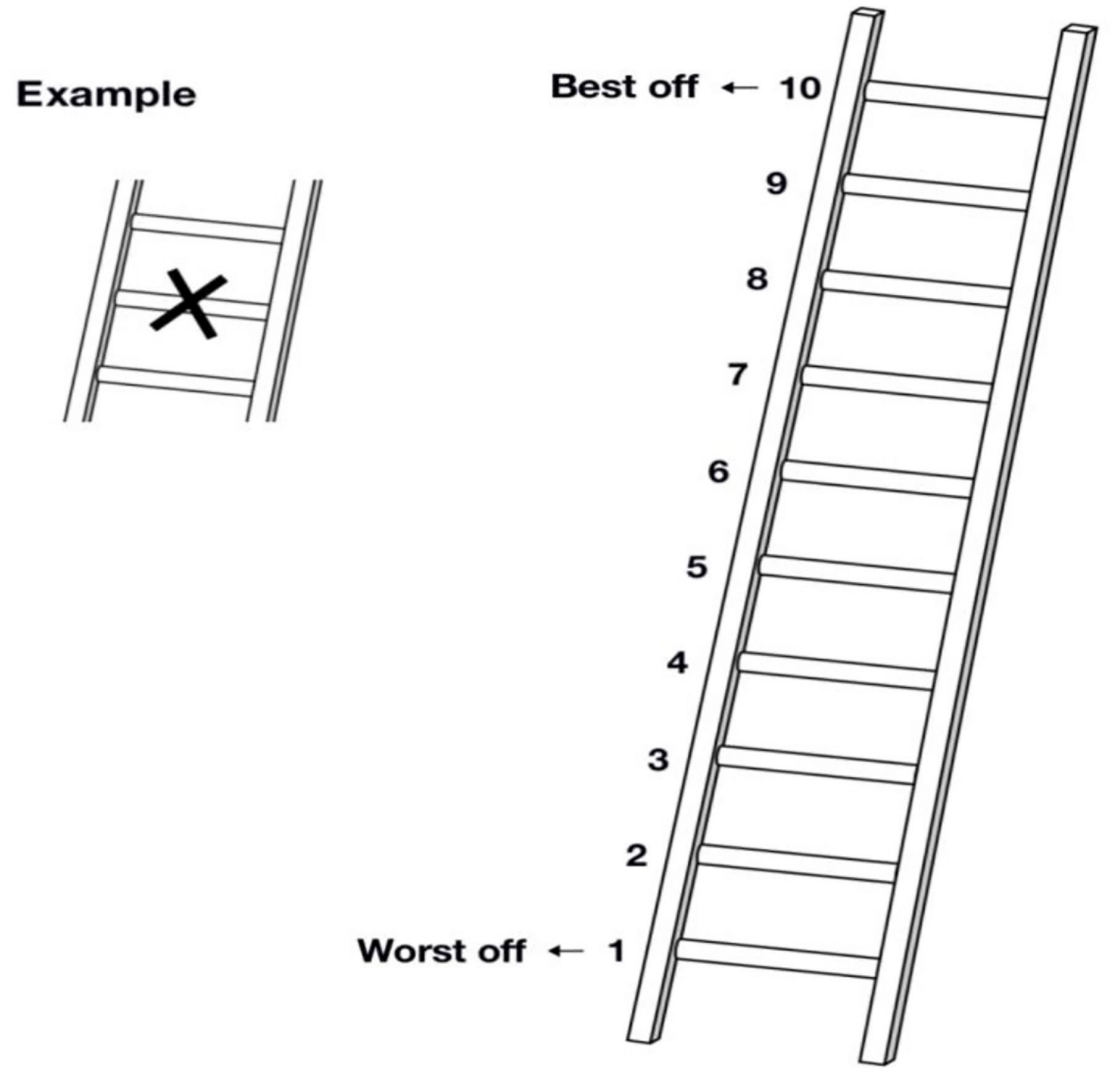
The subjective social status was measured using the MacArthur scale. (1) Here is a ladder. Think of this ladder representing where people stand in Zhejiang Province. At the top of the ladder are the people who are the best off—those who have the most money, the most education, and the most respected jobs. At the bottom are the people who are the worst off—who have the least money, the least education, and the least respected jobs or no jobs. The higher up you are on this ladder, the closer you are to the people at the very top, and the lower you are, the closer you are to the people at the very bottom. If you consider your current situation and compare it with all other people in Zhejiang Province, where would you place yourself on this ladder? Please mark an X on the rung that best represent your situation. (2) Here is another ladder. Think of this ladder as representing where people stand in the people around them. At the top of the ladder are the people who are the best off—those who have the most money, most education, and best jobs. At the bottom are the people who are the worst off—those who have the least money, least education, and worst jobs or no job. If you consider your current situation and compare it with all the people around you, where would you place yourself on this ladder? Please mark an X on the rung that best represent your situation.

Meanwhile, subjective SES can be defined as an individual's common sense perception of their social standing (17, 18). The MacArthur scale was applied to assess the SSS (12, 19–21). The instructions of the MacArthur scale are more complex linguistically speaking because they have long periods and subordinate constructions and thus require substantially greater cognitive skills (22). Based on the previous study (23), we changed the original reference group of these two questions into “Provinces” and “people around” due to the large socioeconomic differences in China's provinces and the unformed concept of “community” in the same sense (Figure 2). Respondents who had put their marks in between two rungs were assigned to the higher levels of these rungs. Each item was counted as 1–10 points, and the total score was 20 points. Higher scores were considered to have higher subjective SES. Based on the scores of 271 participants, subjective SES was divided into two categories with a cutoff point value of 11 such as low subjective SES and high subjective SES. This social status indicator is a well-validated measurement with a strong construct validity and retest reliability (24). We identified a pool of six experts to participate in the content validity evaluation, including three gerontologists and three social science experts. These experts were selected for their extensive experience in gerontology and sociology, respectively. The relevance scale, which inspected the optimal collocation among four different reference groups (“China” and “Community;” “China” and “People around;” “Province” and “Community;” and “Province” and “People around”), along with the introduction, was sent *via* email to these experts. Each of the items was assessed with the following criteria: 1 = not relevant, 2 = somewhat relevant, 3 = quite relevant, and 4 = highly relevant. With the data obtained, we established the content validity index (CVI) for the item level (I-CVI) by dividing the number of experts rated 3 or 4 by the total number of experts. Meanwhile, we established the CVI indicator for the scale level (S-CVI) by summing the number of items rated 3 or 4 and dividing the total number of items. I-CVI reflected the degree of agreement among experts, while S-CVI represented the consensus of all experts. When the number of experts was >5, I-CVI ≥ 0.78 and S-CVI ≥ 0.80 were acceptable. Finally, the subjective SES indicator based on the “Province” and “People around” was selected because it had the best content validity.

#### Dependency Personality Disorder

Dependency personality disorder was assessed by the standardized Minnesota Multiphasic Personality Inventory-II scale. The DPD scale comprised 57 items. The raw score was calculated and converted into a standardized T-score. A score of 60 or above was indicated as the diagnostic criteria for DPD.

#### Cognitive Function

Cognitive function was measured using the Chinese version of the Dementia Assessment Sheet for Community-Based Integrated Care System 21 items (DASC-21) through which we had identified the reliability and validity for elderly people in the Chinese community (25). The DASC-21 comprised two introductory items and 21 assessment items.

#### Social Support

Social support was measured by the Chinese version of the questionnaires of the Older American Resources and Services (OARS). The ratings were summed to yield a total score. High scores indicated high levels of social support.

#### Community Service Resources

Social resources were assessed using four questions as follows: “Does your community have emergency services like an emergency call?,” “Can you get timely treatment when you are seriously ill?,” “Does your community regularly ask about your current condition or do you receive follow-up calls regularly after seeing a doctor?,” and “Do you regularly attend physical examinations?” If respondents responded with “No,” then, the answer was coded as “0;” otherwise, it was coded as “1”.

#### Other Measurements

Depressive symptoms were measured using the 15-item Geriatric Depression Scale. Participants' personality characteristics were assessed by the Eysenck Personality Questionnaire (EPQ).

### Statistical Analysis

Statistical analysis was restricted to the 1,217 participants who had complete questionnaires, objective SES, and DPD assessment data. The demographic characteristics of the participants were described using percentage.

Bivariate analysis for the level of objective SES and related factors was used for the chi-squared test. The participants were divided into two groups, namely, high level of SES and low level of SES.

A logistic regression analysis was performed to identify the association between DPD and objective SES. Individuals with a T-score on the DPD scale ≥60 points were regarded as DPD individuals and expressed as “1;” otherwise, “0” for those score lower than 60 points. As a binary variable, objective SES was divided into high and low groups, and the high group was used as the control group. The logistic regression model was adjusted for age, gender, marital status, chronic disease status, social support, DASC-21, community service resources, GDS-15, and personality.

We conducted the analysis of covariance to evaluate the DPD scores among different SES groups such as high objective SES score and high subjective SES score group (objective high-subjective high), low objective SES score and high subjective SES score group (objective low-subjective high), high objective SES score and low subjective SES score group (objective high-subjective low), and low objective SES score and low subjective SES score group (objective low-subjective low) by using the general linear model procedure with the SAS program PROC glm. A variance homogeneity test and normality test were performed. We calculated the mean and standard error for the DPD score for the four groups, and the linear trend was tested for the means of four groups. Comparisons were conducted among the four groups by using an F test with a significance level of 0.05.

Statistical analysis was performed using SAS for Windows (version 9.4) and statistical package SPSS version 26.0 (IBM Corporation, Armonk, NY, USA). All statistical tests were two-sided with α = 0.05.

## Results

Table 1 shows the demographic characteristics of the study participants. The average age of the participants was 68.5 years. Among all the participants, 486 (39.9%) were men, and 731 (60.1%) were women. Those who lived in rural areas accounted for 58.0 and 42.0% for those in the cities. More than half of the participants had equal or <6 years of schooling or no schooling. A total of 70.5% of participants self-reported having one or more chronic diseases. No statistically significant differences existed in DPD by gender (*p* = 0.234). However, DPD scores increased with age (*p* <0.001). In addition, DPD scores were different in urban (*Mean* = 39.5, *SD* = 11.3) and rural (*Mean* = 44.5, *SD* =12.5) areas (*p* < 0.001).

**Table 1:** Demographic characteristics of the study participants by sex in the study.

**Variables**	**Men (*****N*** **=** **486)**		**Women (*****N*** **=** **731)**	
	***n***	***%***	***n***	***%***
**Age (year)**				
60–69	275	56.6	462	63.2
70–79	186	38.3	223	30.5
≥80	25	5,1	46	6.3
**Education (year)**				
0–6	248	51.0	412	56.3
7–9	132	27.2	173	23.7
10–12	61	12.5	95	13.0
≥13	45	9.3	51	7.0
**Marital status**				
Non-married	44	9.1	183	25.0
Married	442	90.9	548	75.0
**Smoking status**				
Yes	147	30.2	7	1.0
No	339	69.8	724	99.0
**Alcohol use**				
Yes	207	42.6	70	9.6
No	279	57.4	661	90.4
**Physical activity**				
Yes	214	44.0	391	53.5
No	272	56.0	340	46.5
**Individual income**				
¥ 0–1,999	232	47.7	317	43.4
¥ 2,000–3,999	125	25.7	290	39.7
¥ 4,000–5,999	84	17.3	91	12.4
¥ 6,000 and over	45	9.3	33	4.5
**Chronic disease status**				
Yes	353	72.6	505	69.1
No	133	27.4	226	30.9
**Measured variables (Mean, SD)**				
Dependency scores	42.9	11.05	42.1	12.9
SES scores	4.3	2.6	4.1	2.49
GDS-15 scores	2.9	2.9	3.1	3.1
EPQ scores	45.7	8.7	46.2	9.8
DASC-21 scores	27.5	6.3	28.5	7.1

*SES, Socioeconomic Status; GDS, Geriatric Depression Scale; EPQ, Eysenck Personality Questionnaire; DASC-21, Dementia Assessment Sheet for Community-based Integrated Care System 21-items*.

Table 2 shows the results of the levels of objective SES and related risk factors in the chi-squared test. In rural areas, 82.7% had a low level of objective SES (*p* < 0.001). Failure to receive any timely treatment or emergency service had a greater likelihood of a low level of objective SES. The low level of objective SES group compared with the counterpart had a higher proportion of individuals with depressive symptoms (GDS-15 scores ≥ 5, *p* < 0.001) and DPD (T-score ≥ 60, *p* = 0.005).

**Table 2:** The level of socioeconomic for characteristics of participants in chi-squared test.

**Variables**	**Low SES (*****N*** **=** **724)**		**High SES (*****N*** **=** **493)**		***P*-value**
	***n***	***%***	***n***	***%***	
**Region**					
Rural	584	82.7	122	17.3	<0.001
City	140	27.4	371	72.6	
**Age (year)**					
60–69	418	56.7	319	43.3	0.015
≥70	306	63.7	174	36.3	
**Marital status**					
Non-married	151	66.5	76	33.5	0.017
Married	573	57.9	417	42.1	
**Social support**					
≤ 13	394	68.8	179	31.2	<0.001
≥14	330	51.2	314	48.8	
**Timely treatment**					
Yes	692	58.8	485	41.2	0.007
No	32	80.0	8	20.0	
**Emergency service**					
Yes	316	55.4	254	44.6	0.007
No	408	63.1	239	36.9	
**GDS-15**					
0–4	510	54.0	435	46.0	<0.001
≥5	214	78.7	58	21.3	
**Dependency**					
Yes	91	71.1	37	28.9	0.005
No	633	58.1	456	41.9	

*SES, Socioeconomic Status; GDS, Geriatric Depression Scale*.

Table 3 shows the association between the levels of objective SES and DPD status by binary logistic regression analyses after adjusting for depression, personality, community resources related to DPD, cognitive status, social support, and other covariates. The objective SES was significantly negatively associated with the levels of DPD, with odds ratio of 1.84 (95% CI, 1.07–3.18; *p* = 0.028).

**Table 3:** The odd ratios of socioeconomic status for dependency by logistic regression model.

**Variables**	**Dependency status**			
	**OR**	**95% CI**		***P*-value**
Socioeconomic status (High/Low)	1.84	1.07	3.18	0.028
EPQ score	1.14	1.11	1.17	<0.001
GDS-15 (High/Low)	0.29	0.17	0.51	<0.001
Regular physical examination (No/Yes)	0.46	0.26	0.83	0.010
Age (60–69/≥70)	1.45	0.87	2.42	0.154
Gender (Male/Female)	1.42	0.84	2.38	0.188
Marital status (Non-married/Married)	0.99	0.56	1.79	0.993
Chronic disease status (No/Yes)	1.17	0.67	2.05	0.584
Social support (High/Low)	1.19	0.71	2.00	0.518
Timely treatment (No/Yes)	2.08	0.84	5.18	0.115
Regular follow-up (No/Yes)	0.93	0.58	1.50	0.755
DASC-21 score	1.01	0.98	1.05	0.442

*EPQ, Eysenck Personality Questionnaire; GDS, Geriatric Depression Scale; DASC-21, Dementia Assessment Sheet for Community-based Integrated Care System 21-items*.

Table 4 presents the mean DPD scores among different SES groups in 271 participants. The objective high-subjective low group had higher DPD scores than the objective high-subjective high group. The objective low-subjective low group had the highest score among the four groups. Although the mean DPD score of the objective low-subjective high group was higher than the reference group, no statistical significance existed between the two groups. Based on the results of analysis of covariance (ANCOVA), the DPD score means of these groups increased gradually with a significant linear trend (*p* = 0.005). Then, they were adjusted for gender, marital status, chronic disease status, 2-week prevalence, alcohol use, mobile use, DASC-21 points, living spaces, and EPQ.

**Table 4:** The mean and standard error for dependency scores in different groups of SES and SSS by the analysis of covariance.

**Variable categories**	**Dependency**			
	***n***	***Mean***	***SE***	***P*-value**
**Socioeconomic status-subjective social status**				
High-high	83	35.62	1.03	
Low-high	71	37.84	1.13	0.160
High-low	40	39.19	1.52	0.048
Low-low	77	39.29	1.07	0.017
	*P* for trend	0.005		

*Adjusted for gender, marital status, chronic disease status, 2-week prevalence, alcohol use, mobile use, DASC-21 points, living spaces and EPQ. SES, Socioeconomic Status; SSS, Subjective Social Status; DASC-21, Dementia Assessment Sheet for Community-based Integrated Care System 21-items; EPQ, Eysenck Personality Questionnaire*.

## Discussion

This study observed the association between SES and DPD among the elderly. We found that the levels of objective SES were negatively associated with the DPD scores by logistic regression. Further results showed that the subjective SES was more strongly associated with DPD than objective SES.

Our results show that SES is associated with places of residence, emergency services, timely treatment, and social support. The majority of the elderly with low objective SES live in rural areas. The increased risk of DPD at a lower level of objective SES may be due to greater stress exposure and reduced resources that buffer its effects (26). Stress exposure is often invoked as important pathways linking lower objective SES to poorer health (27). At the same time, individuals with fewer social and economic resources have added difficulty in obtaining general medical care information and services, including preventive health services such as screening. Under this state of inequality, they tend to choose emergency departments and small clinics to acquire timely treatment and emergency services, thus observing dependency. A previous study has confirmed that, compared with areas with higher objective SES, areas with lower objective SES have a higher incidence of external cardiac arrest due to insufficient emergency resources, such as automatic external defibrillators, thus contributing to dependency (28). Resources include not only material resources that individuals can obtain and use but also psychosocial resources that are intangible but significant. Compared with people who are not dependent, the feeling of inequality in material and psychosocial resources among dependent people will exacerbate their dependency and drive them to rely on relevant resources to acquire support (29). Gallo et al. (30) observed a significant and moderate correlation between higher levels of objective SES and higher psychosocial resources in middle-aged Mexican American women. People with low objective SES encounter more negative life events and chronic stressors (31). The lack of coping resources, especially psychosocial resources, such as social support, will further lead to adverse effects on individuals with low objective SES (32). In addition, psychosocial resources can operate through cognitive and emotional states, such as self-efficacy and self-control, which are inherently related to DPD (33). Social support has a protective effect on the dependency of the elderly with low levels of objective SES. Research shows that people with larger social networks are also more likely to have an active lifestyle and a better state of health. However, previous research has pointed out that the elderly in a community environment may improve positive connections with other people to reduce loneliness and increase security, which may lead to further dependency. Additional research is needed to determine the dividing point between adaptive and maladaptive dependency in the future.

The association between SES and DPD was also observed by using subjective measurement tools. Regardless of the objective SES, subjective SES has a stable association with DPD. The analysis of association between DPD and different groups of SES showed that those individuals with low objective SES and low subjective SES had the highest DPD scores. Moreover, participants with high levels of objective SES and high perceived social status had the lowest DPD scores. For those individuals with high subjective SES, no statistical significance exists between objective SES and DPD. These results suggest that subjective perceptions of SES have a stronger effect on DPD than objective measurements, and a high perceived social status can reduce the risk of DPD.

Among individuals with high subjective SES, we found that they can be characterized by better self-perceived health, better perceived financial status, and higher self-efficacy compared with their peers (results not shown), resulting in positive evaluation and higher self-satisfaction. Senectitude is a special period of life, and previous achievements and status are no longer important in this period (34). A study reported that well-adjusted elderly people are more resilient in suffering from objective status forfeiture compared with middle-aged adults (35). These individuals are able to accept positive or negative lives and have higher adaptive flexibility in coping with age-related losses and restrictions, achieving the balance between dependency and autonomy. These elderly people who are emotionally adaptive tend to have a low level of dependency. Based on the psychosocial hypothesis, psychological stress is related to adverse perception, which is detrimental to health (36). We can interpret this hypothesis as follows: the elderly people with high subjective SES can reduce DPD through the positive perception of objective conditions, which protects their mental health.

Subjective SES has a stronger correlation with DPD than objective SES, that is, subjective SES can be regarded as the “cognitive average,” which includes the evaluation of education and socioeconomic factors obtained in the past and future development prospects (37). Subjective SES not only evaluates previously obtained traditional measurements, such as education, income, and occupation status, but also assesses the self-perceived esteem and social capital from others (38). Evidence suggests that the subjective assessment of SES at the individual level may be a better assessment than any objective SES indicator.

The majority of these studies focus on the association between subjective SES and health. To the best of our knowledge, few studies have explored the association between subjective SES and DPD. We attempt to clarify the association between subjective SES and DPD by the perception of position in the social hierarchy. This perception will generate cognitive and emotional responses that will mediate the detriment of low subjective SES to DPD. Individuals with higher levels of dependency may be more vulnerable to negative emotions. This DPD is operated by the negative emotion and stress mechanism of psychoneurobiology (39). Given the persistent or recurring negative emotions and stress responses caused by perceived low social status, lower subjective SES may lead to higher risks of DPD (40).

The main strength of this study is that we collect comprehensive data so that we not only can explore the variables that captured our interest but also fully adjusted potential risk factors, including cognition, personality, and depression, eliminating their effect on the association between DPD and subjective and objective SES. In addition, we tested the content validity of the objective SES index to further confirm the validity of the scale. Our study also has several limitations. First, the cross-sectional research design limits us to determine the causality between the SES and DPD. Thus, the complex and changing trends of behavior factors and the risk of DPD over time cannot be evaluated. Although a significant linear trend exists in the four categories of deterrent SES by using GLM to evaluate the DPD scores, we could not assess precisely the cause and effect between DPD and the levels of SES. In further studies, a longitudinal study design is needed to clarify possible causality. Second, owing to the limitations of the sample size of subjective SES, we still need to verify the mechanism of the association between DPD and subjective SES.

## Conclusion

This study determined the association between poor SES and increased risk of DPD, and the results of this work represent preliminary evidence that perceived social status has a stronger association with DPD than objective SES. The effect of subjective SES on DPD is possibly associated with the perception of position in the social hierarchy. However, several questions remain unanswered and represent promising directions for future work. There is abundant room for further progress in determining the pathways of subjective SES and DPD to fully understand the complicated causality and provide targeted support strategies to reduce or delay the dependency of elderly people.

## Data Availability Statement

The datasets used and/or analysed during the current study are available from the corresponding author on reasonable request.

## Ethics Statement

The studies involving human participants were reviewed and approved by all participants provided informed consent before participation. The study was approved by the Institutional Review Board at the School of Public Health, Zhejiang University. The patients/participants provided their written informed consent to participate in this study.

## Author Contributions

YP and YL were the principal investigators and involved in the study design, conception, and manuscript preparation. AA, XD, and YC performed data collection and analysis. All authors contributed to the article and approved the submitted version.

## Funding

This study was supported by the Leading Innovative and Entrepreneur Team Introduction Program of Zhejiang (2019R01007) and in part by the Key Laboratory of Intelligent Preventive Medicine of Zhejiang Province (2020E10004).

## Conflict of Interest

The authors declare that the research was conducted in the absence of any commercial or financial relationships that could be construed as a potential conflict of interest.

## Publisher's Note

All claims expressed in this article are solely those of the authors and do not necessarily represent those of their affiliated organizations, or those of the publisher, the editors and the reviewers. Any product that may be evaluated in this article, or claim that may be made by its manufacturer, is not guaranteed or endorsed by the publisher.
