# Corrigendum: Socioeconomic status association with dependency from objective and subjective assessments: A cross-sectional study

**DOI:** 10.3389/fpsyt.2022.1004126

**Published:** 2022-11-02

**Authors:** YiYang Pan, Ayizuhere Aierken, XiWen Ding, Yuan Chen, Ying Li

**Affiliations:** Department of Social Medicine, School of Public Health, Zhejiang University, Hangzhou, China

**Keywords:** dependency, socioeconomic status, social resources, objective and subjective assessments, elderly people

In the published article, the authors found that identifiable data is included and the entire supplementary material file needs to be removed.

The authors apologize for this error and state that this does not change the scientific conclusions of the article in any way. The original article has been updated.

## Publisher's note

All claims expressed in this article are solely those of the authors and do not necessarily represent those of their affiliated organizations, or those of the publisher, the editors and the reviewers. Any product that may be evaluated in this article, or claim that may be made by its manufacturer, is not guaranteed or endorsed by the publisher.

