# Erratum: Socioeconomic status association with dependency from objective and subjective assessments: A cross-sectional study

**DOI:** 10.3389/fpsyt.2022.1091700

**Published:** 2022-11-15

**Authors:** Frontiers Production Office

**Affiliations:** Frontiers Media SA, Lausanne, Switzerland

**Keywords:** dependency, socioeconomic status, social resources, objective and subjective assessments, elderly people

Due to a production error, the **Data Availability Statement** was incorrectly written as “The original contributions presented in the study are included in the article/Supplementary Material, further inquiries can be directed to the corresponding author.” It should be “The datasets used and/or analysed during the current study are available from the corresponding author on reasonable request.”

The publisher apologizes for this error and states that this does not change the scientific conclusions of the article in any way. The original article has been updated.

## Publisher's note

All claims expressed in this article are solely those of the authors and do not necessarily represent those of their affiliated organizations, or those of the publisher, the editors and the reviewers. Any product that may be evaluated in this article, or claim that may be made by its manufacturer, is not guaranteed or endorsed by the publisher.

